# Assessing the Role of lncRNA GAS5 in Thyroid Function Regulation and Their Potential Use as Biomarkers for Hyperthyroidism Management

**DOI:** 10.12688/f1000research.173609.1

**Published:** 2025-12-23

**Authors:** Yasser A. Muhammad, Mohammed j. Muhaidi

**Affiliations:** 1 University of Fallujah College of Applied Sciences, Al-Fallujah, Al Anbar Governorate, Iraq

**Keywords:** Key words : lncRNAs, GAS5, hyperthyroidism, Gene expression, Thyroid hormones (T3, T4)

## Abstract

Long non-coding RNAs (lncRNAs) are increasingly recognized as important regulators of cellular processes, including those implicated in autoimmune and endocrine disorders. However, their role in thyroid function, particularly hyperthyroidism, remains insufficiently defined. Growth Arrest-Specific 5 (GAS5) is a lncRNA with established functions in tumor suppression, cell cycle arrest, and apoptosis, and may therefore influence thyroid cell regulation and hormone production. This study aimed to assess the expression of GAS5 in patients with hyperthyroidism and evaluate its potential utility as a biomarker. Thirty whole-blood samples from hyperthyroid patients and 20 samples from healthy controls were analyzed. Total RNA was extracted using Trizol, and complementary DNA (CDNA) was synthesized with oligo(dT) primers. GAS5 expression was quantified by qRT-PCR, and statistical analysis was performed using One-Way ANOVA, correlation testing, and visualization in R. GAS5 expression was significantly decreased in hyperthyroid patients compared with controls (mean 0.429 vs. 1.004, *p* < 0.0001), with non-overlapping confidence intervals. Approximately 77% of patient samples showed expression levels below one. Pairwise comparisons confirmed highly significant differences between groups ( *p* = 0.0001). Spearman correlation revealed an inverse relationship between GAS5 and T3 ( *r* = –0.454, *p* = 0.001) as well as T4 ( *r* = –0.438, *p* = 0.001).These findings indicate that GAS5 downregulation is associated with increased thyroid hormone production, suggesting a potential regulatory role in hyperthyroidism. GAS5 expression could therefore represent a promising early biomarker for individuals at risk of thyroid dysfunction.

## Introduction

Hyperthyroidism is among the most prevalent thyroid disorders characterized by excessive thyroid hormone production. It relates to the condition where there is an overactivity of the thyroid gland. The terms hyperthyroidism and thyrotoxicosis are sometimes viewed as having the same meaning. It is a condition of an abnormal and extreme amount of thyroid hormones in the body. ^1^ Hyperthyroidism can either be overt or subclinical. The conditions with TSH levels lower or undetectable and T3 and/or T4 levels higher together with T3 thyrotoxicosis and low or undetectable TSH with normal T4 levels are categorized as T3 toxicosis. The subclinical stage of hyperthyroidism is characterized with TSH levels lower or undetectable and T3 and T4 levels normal. ^2^

This lncRNA is a transcript of the growth arrest-specific 5 (GAS5) gene, a non-protein coding gene, first discovered in 1988 while searching for new suppressors of cancer using subtractive cDNA cloning of genes that are more active in cells that are not growing. ^3^ GAS5 is situated at 1q25.1 and encodes small nucleolar RNAs (snoRNAs), microRNAs (miRNAs), PIWI-interacting RNAs (piRNAs), and long non-coding RNA (lncRNA). ^4– 6^ The gene has 12 exons, which include a short open reading frame and are not thought to code for a functional protein. Instead, these exons are spliced together to make two possible mature lncRNAs, called GAS5 a and GAS5b, because exon 7 has different 5′-splice donor sites. ^6^

Studies involving these systems and unmodified primary cells suggest that GAS5 exerts contradictory effects on cell proliferation (inhibitory) and apoptosis (stimulatory), which together likely comprise the fundamental basis of the tumor suppressor role in vivo. Additional processes associated with cell migration and invasion may contribute to certain malignancies, as GAS5 lncRNA has recently been demonstrated to inhibit the migration of renal cell and cervical cancer cell lines, ^7, 8^ while exhibiting no influence on the migration of NSCLC cells. ^9^

Cyclin-dependent kinase 6 (CDK6), which helps the cell cycle move from G1 to G1/S, is a protein that is linked to GAS5 in bladder carcinoma cells. ^10^ Silencing of GAS5 elevates, while ectopic expression of GAS5 lncRNA diminishes, the mRNA and protein levels of CDK6 in cell lines. A comparable, negative correlation between GAS5 and CDK6 levels has been documented in bladder and pancreatic cancer tissues, indicating that this interaction occurs in vivo. The reduction of CDK6 expression in these cells inhibits the stimulation of cell proliferation resulting from GAS5 silencing; conversely, the overexpression of CDK6 alleviates the suppression of cell proliferation caused by GAS5 lncRNA. The effects are partial. ^10, 11^ The primary importance of our work lies in the anticipated decreased expression of the GAS5 gene in individuals with hyperthyroidism. Given its regulatory function in proliferation and hormone-related signaling, GAS5 may also contribute to the pathophysiology of thyroid disorders, including hyperthyroidism. The Aim of This Research Is Investigate the expression levels of lncRNA GAS5 in the blood of hyperthyroid patients compared to healthy controls by qPCR, Determine the correlation between GAS5 expression and thyroid hormone levels (T3, T4, and TSH) in hyperthyroid patients, Evaluate the potential of GAS5 as a molecular biomarker for thyroid dysfunction

## Methods

### Sample collection

After getting permission from the Iraqi Ministry of Health, and Ramadi Teaching Hospital samples were taken from people with hyperthyroidism (n = 30) and healthy people (n = 20). In an EDTA tube, 200 microliters of blood were mixed with 600 microliters of Trizol. The sample collection period was from 2025/8/10 to 2025/9/20.

### RNA extraction

According to the company’s protocol (Geneaid company, Taiwan) https://www.geneaid.com/Tri-RNA/GZXD total RNA was extracted from white blood cells.

### Add poly-A tail

To enable the detection of ncRNA molecules by quantitative reverse transcription PCR (qRT-PCR), an artificial polyadenylation step was performed, as these transcripts naturally lack a poly(A) tail. Briefly, 10 μL of total RNA extracted from blood samples was transferred into a sterile PCR tube. The reaction mixture was prepared by adding 2 μL of Poly(A) buffer, 1 μL of ATP, and 1 μL of Poly(A) polymerase to each sample. The tubes were then incubated in a thermal cycler at 42 °C for 45 minutes, followed by 85 °C for 15 seconds to inactivate the enzyme, in a single cycle. The polyadenylated RNA was subsequently used as a template for reverse transcription with an oligo(dT) primer, prior to downstream qRT-PCR analysis.

### Synthesis complementary DNA (cDNA)

Complementary DNA (cDNA) was synthesized using a ready-to-use cDNA synthesis kit (Bioneer, Korea) ( https://eng.bioneer.com/20-k-2201-cfg.html) following the manufacturer’s protocol with minor adjustments. Briefly, 10 μL of RNA extract from each sample was transferred into a sterile microfuge tube. To this, 4 μL of cDNA master mix containing oligo(dT) primers (specific for eukaryotic transcripts) was added, followed by 6 μL of RNase-free water. The reaction mixture was gently mixed and incubated in a thermal cycler at 37 °C for 15 minutes to allow reverse transcription, followed by enzyme inactivation at 85 °C for 15 seconds in a single cycle. The synthesized cDNA was either used immediately as a template for qRT-PCR or stored at −20 °C for later use.

### Primer design

The gene sequence was imported from NCBI ( https://www.ncbi.nlm.nih.gov/nuccore/NC_000001.11?report=fasta&from=173863901&to=173869045&strand=true) using Primer3Plus software ( https://www.primer3plus.com/index.html). The primer for the GAS5 gene was designed as GAS5 F GAAGCCCCTGGAGGAAAGTC GAS5 R GCCTTGTCCCCATCTTCTCC by and GAPDH, forward, 5′-GGTCTCCTCTGACTTCAACA-3′; reverse, 5′-GTGAGGGTCTCTCTCTTCCT-3′ (Macrogen, Korea).

### Quantitative reverse transcription PCR (qRT-PCR)

Quantitative reverse transcription PCR (qRT-PCR) was performed to assess gene expression levels. For each reaction, 2 μL of synthesized cDNA was transferred into a PCR tube, followed by the addition of 0.5 μL of each primer and 10 μL of SYBR Green master mix (Genomic Company). The final reaction volume was adjusted to 20 μL using DNase-free distilled water, and the contents were gently mixed before being loaded into the qPCR system. The amplification protocol included an initial denaturation step at 95 °C for 3 minutes, followed by 40 cycles of denaturation at 95 °C for 15 seconds, annealing at 60 °C for 30 seconds, and extension at 72 °C for 30 seconds. Relative gene expression levels were calculated using the 2^−ΔΔCt method, normalizing each target gene to the corresponding housekeeping gene(GADPH) and comparing samples with their respective controls. Livak equation was used to evaluate the fold expression against house keeping gene and control by the following steps: ^12^

Ct Control – Ct house keeping control = ΔCt control

Ct sample – Ct house keeping sample = ΔCt sample

Δ Ct sample – Δ Ct control = ΔΔ Ct

Fold of gene expression = (2¯ΔΔCt )

### Data analysis

The Statistical Product and Service Solutions SPSS software, version 26 (2019), was used to analyze the results using One-Way ANOVA and Correlations. The R program, version 4.4.1, was used forming figure.

## Result

Table 1 presents the laboratory measurements of the control group, including TSH, T3, and T4 levels, as well as age and BMI, to confirm that all participants were clinically healthy and free of thyroid dysfunction.

**Table 1.** Represents samples of healthy people who do not suffer from hypothyroidism (control). T4, T3 and TSH tests were performed to confirm their validity.

Sample	Age	BMI	T4 (μg/dL)	T3 (ng/ml)	TSH
1	45	24.56747	7.65	1	4.4
2	50	23.30109	7.236	1.19	1.74
3	57	25.78125	10.08	1.193	1.3
4	55	23.87511	5.646	1.051	1.651
5	32	23.05456	7.784	1.095	1.432
6	44	23.33547	9.087	1.681	2.126
7	52	23.24341	8.318	1.133	1.756
8	27	26.02758	8.502	1.357	0.6
9	30	23.93948	6.983	1.235	1.72
10	22	22.67574	7.881	1.409	3.639
11	48	22.85714	6.986	1.323	4.118
12	39	21.77384	7.236	0.984	2.352
13	22	22.20633	7.579	1.077	2.094
14	54	25.55933	7.575	1.113	3.309
15	33	24.97399	6.2	1	2.97
16	37	26.10656	8.069	1.228	1.67
17	26	27.05515	5.587	1.005	1.131
18	50	25.87606	7.48	1.285	2.152
19	31	25.60554	6.45	1.205	0.523
20	29	25.14861	7.78	1.205	1.738

Table 2 shows the clinical results of hyperthyroid patients, where markedly elevated T3 and T4 values and severely suppressed TSH levels confirm the clinical diagnosis of hyperthyroidism.

**Table 2.** Shows samples of patients with hyperthyroidism with T4, T3, and TSH tests.

Sample	Age	BMI	T4 ug\dl	T3 ng\ml	TSH
1	36	26.53376	15.2	2.352	0.005
2	45	34.15978	8.979	1.359	0.014
3	58	24.4646	14.2	2.025	5.223
4	60	25.46198	9.045	1.802	0.05
5	66	19.69267	14.8	2.46	0.01
6	29	22.31328	15.2	0.87	0.01
7	36	23.91883	14.99	3.22	0.02
8	44	20.51913	16.22	3.33	0.12
9	55	26.7094	14.32	4.01	0.05
10	42	20.28123	18	4.11	0.001
11	39	20.2449	18.2	3.31	0.005
12	60	28.57796	15.13	4.91	0.049
13	70	34.375	13.52	4.08	0.102
14	55	25.28257	19.33	4.49	0.048
15	53	37.10938	14.81	4.61	0.032
16	52	29.7442	18.88	4.26	0.01
17	49	28.54828	17.9	4.33	0.011
18	42	31.2213	23	4	0.05
19	51	30.44983	22.5	4.2	0.062
20	34	25.81663	17	4.6	0.005
21	30	25.95156	18.11	5.3	0.003
22	37	28.04282	16.77	4.32	0.005
23	38	21.04805	17	4.22	0.004
24	52	27.47138	12.88	2.33	0.005
25	47	29.41076	12.68	2.46	0.005
26	32	24.67702	12.6	2.7	0.005
27	37	24.60938	13	2.4	0.004
28	35	24.91349	12.7	1.9	0.13
29	44	17.50639	20	5	0.005
30	50	19.15158	19.7	4.94	0.01

Table 3 summarizes the GAS5 gene expression results relative to the GAPDH housekeeping gene, including ΔCt, ΔΔCt, and fold-change values. The results show a clear decrease in GAS5 expression in the majority of patient samples compared with the control group.

**Table 3.** Shows the gene expression of the control and diseased samples.

Sample	CT lncRNA GAS5	CT HK	ΔCTE	ΔCTC	ΔΔCt	Expression Fold Change 2^-ΔΔCt
Control	33.11	30.49	0	2.62	0	1
1	35.27	29.41	5.86	2.62	3.24	0.105843
2	35.92	29.4	6.52	2.62	3.9	0.066986
3	39.36	32.05	7.31	2.62	4.69	0.038741
4	33.83	30.5	3.33	2.62	0.71	0.61132
5	34.92	29.7	5.22	2.62	2.6	0.164938
6	35.73	29.64	6.09	2.62	3.47	0.090246
7	32.87	28.18	4.69	2.62	2.07	0.238159
8	38.99	27.29	11.7	2.62	9.08	0.001848
9	34.94	29.52	5.42	2.62	2.8	0.143587
10	36.73	30.15	6.58	2.62	3.96	0.064257
11	36.88	30.2	6.68	2.62	4.06	0.059954
12	36.12	30.05	6.07	2.62	3.45	0.091505
13	35.26	29.73	5.53	2.62	2.91	0.133046
14	32.18	29.26	2.92	2.62	0.3	0.812252
15	35.09	29.18	5.91	2.62	3.29	0.102238
16	36.3	30.47	5.83	2.62	3.21	0.108067
17	35.77	29.85	5.92	2.62	3.3	0.101532
18	32.39	29.86	2.53	2.62	-0.09	1.06437
19	32.5	30.46	2.04	2.62	-0.58	1.494849
20	35.09	30.25	4.84	2.62	2.22	0.214641
21	28.27	25.6	2.67	2.62	0.05	0.965936
22	36.45	30.04	6.41	2.62	3.79	0.072293
23	33.55	29.62	3.93	2.62	1.31	0.403321
24	29.17	27.59	1.58	2.62	-1.04	2.056228
25	32.84	29.85	2.99	2.62	0.37	0.773782
26	33.33	30.18	3.15	2.62	0.53	0.692555
27	31.45	28.36	3.09	2.62	0.47	0.721965
28	34.53	30.81	3.72	2.62	1.1	0.466516
29	33.17	29.63	3.54	2.62	0.92	0.528509
30	32.75	29.31	3.44	2.62	0.82	0.566442

This table presents the results of a relative gene expression analysis for the long non-coding RNA GAS5, comparing experimental samples to a robustly defined control baseline. The control value (ΔCTC) was not derived from a single measurement but was statistically established from a cohort of 20 control samples as Table 1, whose data were used to calculate a reliable trend line (slope), which was then applied universally across all experimental samples. This approach enhances the statistical validity of the baseline. The subsequent columns detail the standard computational methodology, leading to the calculation of the Fold Change (2^(-ΔΔCt)). Analysis of the final values conclusively demonstrates that the expression of lncRNA GAS5 is significantly suppressed in the vast majority of experimental samples. This conclusion is supported by the fact that 23 out of the 30 samples (approximately 77%) exhibit a Fold Change of less than 1, with a significant proportion showing a severe reduction. The use of a standardized control baseline derived from a larger sample set strengthens the evidence for a prevalent and marked downregulation of lncRNA GAS5 under the investigated conditions.

Table 4 displays the One-Way ANOVA results, which reveal statistically significant differences between the control and patient groups for GAS5 expression and all thyroid hormone parameters (TSH, T3, and T4).

**Table 4.** Analysis of variance (ANOVA, between-subjects effects) indicated that status (control vs patient).

Source	Dependent variable	Type III sum of squares	df	Mean square	F	Sig.
Status	GAS5	3.557	1	3.557	24.308	.0001
	TSH	43.207	1	43.207	44.409	.0001
	T3 ng\ml	52.806	1	52.806	56.110	.0001
	T4 ug\dl	799.232	1	799.232	108.886	.0001

The analysis of variance (ANOVA, between-subjects effects) indicated that Status (control vs patient) had a statistically significant effect on all dependent variables. Specifically, significant main effects were observed for GAS5 (F(1,46) = 24.31, p < 0.0001), TSH (F(1,46) = 44.41, p < 0.0001), T3 (F(1,46) = 56.11, p < 0.0001), and T4 (F(1,46) = 108.89, p < 0.0001). These findings confirm that disease status strongly influenced gene expression (GAS5) and thyroid hormone levels (TSH, T3, T4).

Table 5 presents the adjusted estimated marginal means for GAS5 and thyroid hormones after controlling for age and BMI, demonstrating clear differences between the two groups.

**Table 5.** Estimates for GAS5 expression and thyroid hormone for patients and control.

Dependent variable	Status	Mean	Std. error	95% Confidence Interval	
				Lower bound	Upper bound
GAS5	C	1.004 ^a^	.088	.826	1.182
	p	.429 ^a^	.071	.285	.573
TSH	C	2.172 ^a^	.228	1.713	2.631
	p	.168 ^a^	.184	-.203	.539
T3 ng\ml	C	1.228 ^a^	.224	.776	1.679
	p	3.443 ^a^	.181	3.079	3.808
T4 ug\dl	C	7.365 ^a^	.626	6.104	8.625
	p	15.983 ^a^	.506	14.964	17.001

^a^ Covariates appearing in the model are evaluated at the following values: BMI = 25.3034, Age(year) = 43.2200.

Adjusted estimated marginal means (controlling for age and BMI) further illustrated the differences between groups. In the control group, the mean values were GAS5 = 1.004 (SE = 0.088, 95% CI [0.826–1.182]) and TSH = 2.172 (SE = 0.228, 95% CI [1.713–2.631]), compared with patients who showed lower values (GAS5 = 0.429, SE = 0.071, 95% CI [0.285–0.573]; TSH = 0.168, SE = 0.184, 95% CI [−0.203–0.539]). In contrast, thyroid hormones were markedly elevated in patients compared with controls: T3 = 3.443 (SE = 0.181, 95% CI [3.079–3.808]) and T4 = 15.983 (SE = 0.506, 95% CI [14.964–17.001]), versus controls (T3 = 1.228, SE = 0.224, 95% CI [0.776–1.679]; T4 = 7.365, SE = 0.626, 95% CI [6.104–8.625]).

Table 6 illustrates the pairwise comparisons between the control and patient groups, confirming significant differences in all studied variables.

**Table 6.** Show GAS5 expression and thyroid hormone for patients and control.

Pairwise Comparisons							
Dependent variable	(I) Status	(J) Status	Mean difference (I-J)	Std. error	Sig. ^b^	95% Confidence Interval for difference	
						Lower bound	Upper bound
GAS5	C	p	.575 ^*^	.117	.0001	.340	.810
TSH	C	p	2.004 ^*^	.301	.0001	1.399	2.609
T3 ng\ml	C	p	-2.215 ^*^	.296	.0001	-2.811	-1.620
T4 ug\dl	C	p	-8.618 ^*^	.826	.0001	-10.281	-6.956

Based on estimated marginal means.

* The mean difference is significant at the .05 level.

^b^ Adjustment for multiple comparisons: Least Significant Difference (equivalent to no adjustments).

After adjusting for age and body mass index (BMI), pairwise comparisons between the control group (C) and the patient group (p) revealed highly significant differences across all investigated variables. GAS5 levels were higher in controls compared with patients (Mean Difference = 0.575, SE = 0.117, 95% CI [0.340–0.810], p < 0.0001), as well as TSH (Mean Difference = 2.004, SE = 0.301, 95% CI [1.399–2.609], p < 0.0001). In contrast, T3 and T4 levels were significantly higher in patients compared with controls (T3: Mean Difference = −2.215, SE = 0.296, 95% CI [−2.811–−1.620], p < 0.0001; T4: Mean Difference = −8.618, SE = 0.826, 95% CI [−10.281–−6.956], p < 0.0001). These findings indicate marked differences between the two groups in gene expression (GAS5) and thyroid hormones (TSH, T3, T4).

**Figure 1.**
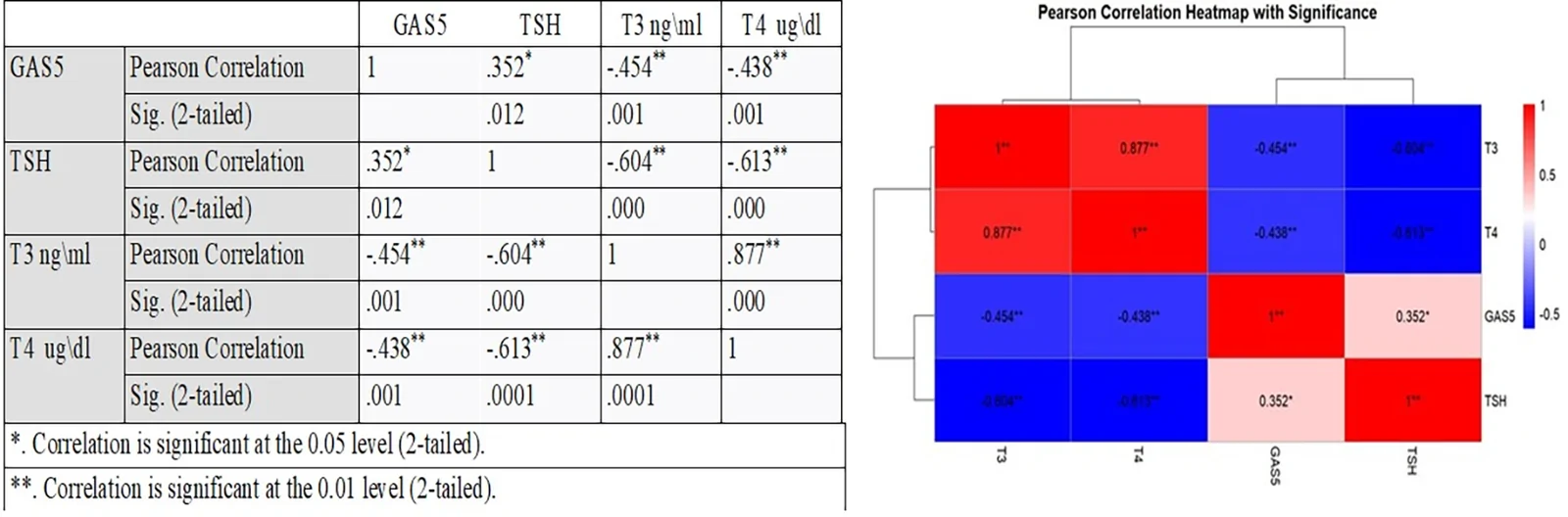
Pearson correlation heatmap showing the relationship between GAS5 expression and thyroid hormones (TSH, T3, and T4). The results demonstrated a significant positive correlation between GAS5 and TSH (r = 0.352, p = 0.012), indicating that higher GAS5 expression is associated with increased TSH levels. Conversely, GAS5 showed a significant negative correlation with both T3 (r = –0.454, p = 0.001) and T4 (r = –0.438, p = 0.001), suggesting that reduced GAS5 expression accompanies elevated thyroid hormone concentrations. Furthermore, a strong positive correlation was observed between T3 and T4 (r = 0.877, p < 0.001), while both hormones were negatively correlated with TSH (r = –0.604 and r = –0.613, respectively; p < 0.001). These findings, also reflected in the heatmap, indicate that GAS5 expression follows a pattern similar to TSH but inversely related to peripheral thyroid hormones (T3 and T4), figure forming by R software.

As shown in Figure 1, the Pearson correlation analysis demonstrates a positive correlation between GAS5 expression and TSH, while GAS5 shows a significant negative correlation with both T3 and T4 levels.

**Figure 2.**
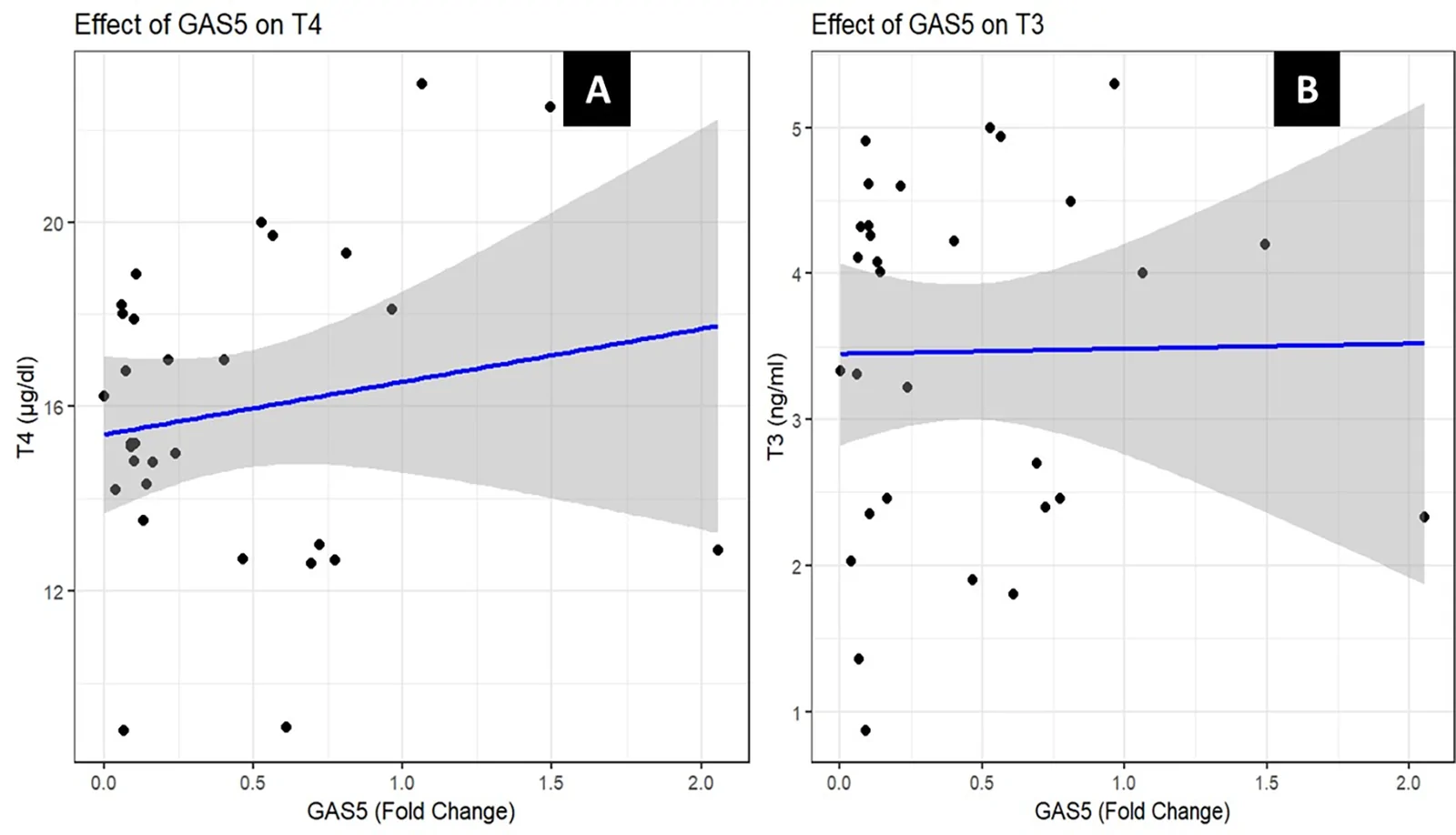
Linear regression plots showing the association between GAS5 expression and thyroid hormones T3 and T4. Correlation and regression analyses were carried out to explore the link between GAS5 expression and thyroid hormones T3 and T4. Pearson’s correlation test showed a significant negative relationship between GAS5 and both T3 (r = –0.454, p = 0.001) and T4 (r = –0.438, p = 0.001). This suggests that as GAS5 levels increase, T3 and T4 concentrations tend to decrease. However, when simple linear regression models were applied, they didn't show any statistically significant effects. For T3, the regression coefficient was β = 0.006 (95% CI: –0.148 to 0.159, p = 0.942, R² = 0.000), and for T4, it was β = 0.024 (95% CI: –0.031 to 0.079, p = 0.383, R² = 0.027). These findings imply that while there's a negative association according to correlation analysis, the actual linear predictive power between GAS5 and thyroid hormones is weak and not statistically meaningful, figure forming by R software.

Figure 2 presents the linear regression analysis evaluating the effect of GAS5 expression on T3 and T4 levels. Although correlation analysis indicated negative associations, the linear regression models did not show statistically significant predictive power.

## Discussion

Our results, consistent with studies on untranslated primary cells as Cao S et al. 2014 and Qiao HP et al. 2013, confirm that the GAS5 lncRNA functions as a tumor suppressor primarily through a fundamental dual mechanism: it simultaneously inhibits cell proliferation and stimulates apoptosis. This opposing action on key cellular processes likely constitutes the principal foundation of its tumor-suppressive activity *in vivo.* Furthermore, supplementary mechanisms involving the inhibition of cell migration and invasion, as observed in renal and cervical carcinoma lines, may contribute to its function in specific cancer contexts. ^7, 8^ This gene may prevent thyroid cells from dividing and producing T4 or T3 hormones. Therefore, our study showed a clear decrease in the expression of this gene in people who suffer from hyperthyroidism so T4 and T3 was high level in our sample.

The inverse association between the GAS5 gene and thyroid hormone levels T3 (r = −0.454, p = 0.0001) and T4 (r = −0.438, p = 0.0001) occurs because this gene affects cell division through its effect on CDK6 proteins. A key mechanism through which the tumor-suppressive effects of GAS5 lncRNA are exerted was identified by Liu Z et al 2013 and Lu X et al 2013 the direct negative regulation of CDK6. Specifically, CDK6 levels were elevated by GAS5 silencing and reduced by its overexpression, at both the mRNA and protein levels. Crucially, this inverse correlation was also observed in clinical samples from bladder and pancreatic cancers, confirming its relevance in vivo. By functional rescue experiments, it was demonstrated that the pro-proliferative effect of GAS5 knockdown was mitigated by CDK6 reduction, whereas the anti-proliferative effect of GAS5 was partially reversed by CDK6 overexpression. Thus, the downregulation of CDK6 is positioned as a primary, though partial, mediator of the ability of GAS5 to inhibit cell proliferation. ^10, 11^ This explains the decreased expression of the GAS5 gene in patients with hyperthyroidism.

Our research indicates that the GAS5 gene may serve as a biomarker for individuals with hyperthyroidism. This finding is based on numerous prior research that identified reduced gene expression of this gene as a biomarker in various organs, including mesothelioma, prostate cancer, and esophageal cancer. Renganathan et al. (2014) examined the reduced expression of GAS5 using primary mesothelioma cell cultures. ^13^ Although its major role is identified as a tumor suppressor, GAS5 may potentially act as an oncogene, not only in mesothelioma but also in other malignancies, including prostate and esophageal tumors. ^14, 15^ To our knowledge, this is the first investigation utilizing circulating GAS5 as a diagnostic marker for the identification of malignant mesothelioma through liquid biopsies. Kresoja-Rakic et al identified GAS5 as a predictive factor by analyzing plasma samples from mesothelioma patients prior to and following chemotherapy. ^16^

Furthermore, despite the fact that our correlation analysis indicated a strong negative association of GAS5 with thyroid hormones T3 and T4, the linear regression analysis results do not support this statistically. This might be an indication that the relationships between GAS5 and thyroid hormones are not simple or nonlinear. According to GAS5 might have a complex effect, possibly in a multi-step way, on the transcription of regulatory mechanisms with or without affecting the final mRNA or maturation efficiency of certain mRNA. GAS5, however, might not target the TSH–T3/T4 system itself but rather indirectly effect it for example activation of hormone receptors when TSH is low or influence the transcription factors linked to T3/T4 levels signaling. Thus, GAS5 regulates thyroid hormone levels in a nonlinear, complex manner and this cannot be simply explained by the direct simple reverse correlation between mRNA expression level and hormone level concentrations in the blood.

One of the problems and limitations that we faced in our study is the sample size, as 30 samples are barely representative of the community, as well as the time taken to collect samples. We also faced some delays in the work due to international shipping of materials imported from the companies mentioned above. This study confirms the importance of early genetic detection of the GAS5 gene, which may help in early diagnosis of people suffering from primary hyperthyroidism, which helps in early treatment and prevents the symptoms from worsening or leading to an enlargement of the thyroid gland, which in advanced stages may turn into cancer.

We recommend and emphasize measuring gene expression in patients with hyperthyroidism before and after chemotherapy with radioactive iodine. We also recommend that the sample size be greater than 30.

## Ethical considerations

This study was conducted in accordance with the Declaration of Helsinki. Ethical approval was obtained from the Anbar Health Directorate / Iraqi Ministry of Health (Institutional Review Board – IRB) under approval number: **[2025035]**.

The committee reviewed and approved all procedures related to human sample collection prior to the initiation of the study.

## Informed consent

Verbal informed consent was obtained from all participants prior to blood sample collection. Written consent was not required because the study involved minimal-risk procedures (non-invasive blood sampling) and no identifiable personal information was collected, in accordance with the ethical guidelines approved by the Anbar Health Directorate IRB.

## Data Availability

All underlying data supporting the findings of this study—including raw Ct values for GAS5 and GAPDH, ΔCt and ΔΔCt calculations, fold-change expression values, and all clinical parameters (TSH, T3, T4, BMI, and age)—are openly available in Figshare under a Creative Commons Attribution 4.0 International (CC BY 4.0) license. The complete dataset can be accessed at the following DOI: https://doi.org/10.6084/m9.figshare.30858434. ^17^ All data are fully anonymized and contain no personally identifiable information.
