# Supplementary material for: Assessing the Role of lncRNA GAS5 in Thyroid Function Regulation and Their Potential Use as Biomarkers for Hyperthyroidism Management
Source: F1000Res. 2025 Dec 23;14:1436. [Version 1] doi: 10.12688/f1000research.173609.1 (PMC13560045; doi:10.12688/f1000research.173609.1)
Supplement: Peer review history

# Reviewer report: Reviewer response for version 1

**DOI:** 10.5256/f1000research.191439.r492048

**Published:** 2026-07-06

**Authors:** Hasan Falah Lahij

**Affiliations:** 1 Almaarif University College, Ramadi, Iraq; 2 Biology, University of Al-Maarif, Ramadi, Iraq

The article titled "Assessing the Role of lncRNA GAS5 in Thyroid Function Regulation and Their Potential Use as Biomarkers for Hyperthyroidism Management" investigates the altered expression of the long non-coding RNA Growth Arrest-Specific 5 (lncRNA GAS5) in individuals diagnosed with hyperthyroidism. Using a case-control cohort comprising 30 hyperthyroid patients and 20 healthy clinical controls, the authors extracted total RNA from whole blood using TRIzol, performed artificial polyadenylation, synthesized cDNA, and quantified expression levels via qRT-PCR normalized to the GAPDH housekeeping gene.

 Statistical and clinical findings reveal a marked decrease in GAS5 levels in hyperthyroid patients compared to the baseline level of the control group (mean expression 0.429 vs. 1.004, p<0.0001), with approximately 77% of patient samples showing a significant decrease in GAS5 expression to below 1. Furthermore, correlation analyses demonstrated a statistically significant inverse relationship between circulating GAS5 levels and peripheral thyroid hormones T3 (r = -0.454) and T4 (r = -0.438), mirroring the expected positive trend with TSH. While the linear regression model showed weak predictive power suggesting more complex, multi-step, or nonlinear downstream regulatory pathways the experimental data conclusively link decreased GAS5 levels with overproduction of thyroid hormones.

**Relevance of the Work:**

 The relevance and merit of this research lie in its novel focus on circulating non-coding RNA dynamics within endocrine and metabolic disorders, particularly hyperthyroidism, which has been historically understudied compared to oncological applications. Epigenetic regulators like lncRNAs offer invaluable insights into cellular signaling pathways, such as the speculative involvement of the GAS5-CDK6 cell-proliferation axis mentioned by the authors. Identifying non-invasive molecular biomarkers via liquid biopsies is crucial for the advancement of clinical serology and early diagnostic frameworks. If fully validated, tracking circulating GAS5 could provide clinical laboratories with an early genetic warning system for patients at risk of worsening primary thyroid dysfunction or related cellular hyperplasia. This makes the manuscript highly relevant to the scope of molecular biology, endocrinology, and contemporary translational medicine.

**Second: Detail about the strengths and weaknesses of the article**

**Strengths of the Article:**

 1-sNovelty and Clinical Relevance: Investigating circulating lncRNAs in thyroid dysfunction is a highly relevant and emerging field. While lncRNA GAS5 has been heavily studied in oncology, its expression profile in metabolic and autoimmune endocrine disorders like hyperthyroidism remains largely unexplored, making this a potentially novel contribution.

 2- Methodological Transparency: The molecular workflow is clearly structured and replicable. The authors explicitly detail their RNA extraction, the necessary artificial polyadenylation step (since lncRNAs lack natural poly-A tails), cDNA synthesis parameters, and the quantitative framework using the standard Livak

 3- Accurate baseline determination: Instead of comparing the experimental samples to a single random control sample, the control baseline (ΔC_t) was statistically derived from a well-defined cohort of 20 healthy individuals. This multi-sample standardization enhances the statistical validity of the baseline trend.

 4- Open Data Practices: In alignment with modern open-science mandates, the authors made their complete, anonymized dataset openly accessible via Figshare, ensuring full reproducibility of their Ct calculations and clinical metrics.

**Weaknesses and Areas for Improvement**

1. Unsubstantiated Biomarker Claims: The authors heavily advocate for lncRNA GAS5 as a clinical biomarker for hyperthyroidism. However, they do not provide a Receiver Operating Characteristic (ROC) curve analysis or report the Area Under the Curve (AUC), sensitivity, or specificity. Without these diagnostics, declaring a gene as a potential biomarker based solely on a correlation is premature.
2. Typographical and Nomenclature Errors: There are recurrent typographical errors in vital scientific nomenclature across the text—most notably miswriting the crucial housekeeping gene as "GADPH" instead of the universally accepted GAPDH.
3. Small and Homogeneous Sample Size: As acknowledged by the authors, a cohort of 30 patients and 20 controls is quite small. Furthermore, the power of the study is weakened by the lack of demographic or clinical subgrouping (e.g., separating results by biological sex, age brackets, or specific hyperthyroid etiologies like Graves' disease vs. toxic multinodular goiter).

Is the work clearly and accurately presented and does it cite the current literature?

Yes

If applicable, is the statistical analysis and its interpretation appropriate?

Yes

Are all the source data underlying the results available to ensure full reproducibility?

Yes

Is the study design appropriate and is the work technically sound?

Yes

Are the conclusions drawn adequately supported by the results?

Yes

Are sufficient details of methods and analysis provided to allow replication by others?

Partly

Reviewer Expertise:

Molecular biology

I confirm that I have read this submission and believe that I have an appropriate level of expertise to confirm that it is of an acceptable scientific standard, however I have significant reservations, as outlined above.

# Response

**Published:** 2026-07-26

**Authors:** yaser ammar

**Affiliations:** biotechnology, University of Fallujah, Anbar/ Fallujah, Iraq

We sincerely thank the reviewer for their constructive comments and valuable insights regarding our manuscript titled **"Assessing the Role of lncRNA GAS5 in Thyroid Function Regulation and Their Potential Use as Biomarkers for Hyperthyroidism Management"**. We have carefully addressed all the points raised and revised the manuscript accordingly to significantly improve its scientific quality and clarity.

**Comment 1:** "The authors conclude that GAS5 is an early biomarker without using the ROC curve, AUC, or sensitivity testing. If you need to claim that GAS5 is a biomarker, you must provide an ROC curve analysis and a statement of sensitivity and specificity."

**Author's Response:**

 We performed the ROC test and found high diagnostic power for GAS5 in relation to the disease. The table and figure have been added in the revised version.

**Comment 2:** "There are spelling errors throughout the manuscript, such as 'GADPH' being written instead of 'GAPDH'."

**Author's Response:**

 We apologize for these typographical errors. The entire manuscript has undergone rigorous line-by-line proofreading. All spelling and formatting errors have been corrected, including correcting 'GADPH' to 'GAPDH' throughout the text and tables.

**Comment 3:** "The sample size is small, which weakens the biomarker's strength." Try creating demographic subgroups (i.e., distinguishing between males and females as a demographic criterion) to strengthen your findings.

**Author's response: **We agree with the reviewer that the sample size is small, consisting of 30 patients and 20 healthy individuals. This was explicitly stated in the limitations section, and we recommended that future studies be more comprehensive in terms of sample size. However, in measuring gene expression, we believe this may be sufficient or a starting point for future research.

# Reviewer report: Reviewer response for version 1

**DOI:** 10.5256/f1000research.191439.r461844

**Published:** 2026-03-02

**Authors:** Ahmed AbdulJabbar Suleiman

**Affiliations:** 1 University of Anbar, Al-Anbar, Iraq

**This study investigates circulating lncRNA GAS5 expression in hyperthyroid patients and evaluates its correlation with thyroid hormone levels (T3, T4, TSH). The topic is relevant and potentially novel, as limited studies have explored circulating lncRNAs in thyroid dysfunction. The manuscript is generally structured well; however, several methodological, statistical, and conceptual issues need clarification before the study can be considered robust.**

**1-**The mechanistic link between GAS5–CDK6 axis and thyroid hormone synthesis is speculative and not experimentally tested , you say that this axis has a correlation with thyroid hormone balance and this not proved in your result , so such an important note must be really proved. 

 2-Sample size is small which weakens the power of biomarkers. Try to make demographic subgroups (i.e make male and female differentiation as demographic criteria ) to support your result power.  

 3- Authors conclude that GAS5 is an early biomarker without ROC, AUC, sensitivity test. If you need to say that  GAS5 is biomarker you must but ROC analysis and give us sensitivity and specificity 

 4-There is an word errors across all manuscript such as "GADPH not GAPDH"

Is the work clearly and accurately presented and does it cite the current literature?

Yes

If applicable, is the statistical analysis and its interpretation appropriate?

Yes

Are all the source data underlying the results available to ensure full reproducibility?

Yes

Is the study design appropriate and is the work technically sound?

Yes

Are the conclusions drawn adequately supported by the results?

Yes

Are sufficient details of methods and analysis provided to allow replication by others?

Yes

Reviewer Expertise:

Biotechnology

I confirm that I have read this submission and believe that I have an appropriate level of expertise to confirm that it is of an acceptable scientific standard.

# Response

**Published:** 2026-07-26

**Authors:** yaser ammar

**Affiliations:** biotechnology, University of Fallujah, Anbar/ Fallujah, Iraq

We sincerely thank the reviewer for their constructive comments and valuable insights regarding our manuscript titled **"Assessing the Role of lncRNA GAS5 in Thyroid Function Regulation and Their Potential Use as Biomarkers for Hyperthyroidism Management"**. We have carefully addressed all the points raised and revised the manuscript accordingly to significantly improve its scientific quality and clarity.

 Below is our point-by-point response to each comment:

 Comment 1: “The mechanistic link between the GAS5–CDK6 axis and thyroid hormone synthesis is a hypothetical link that has not been experimentally tested. You stated that this axis is involved in thyroid hormone homeostasis, and this was not demonstrated in your results, so this important observation needs to be confirmed.”

 Author's Response:

 We fully agree with the reviewer's critical assessment. Our current study was designed as an observational clinical trial to evaluate serum GAS5 lncRNA expression levels and their relationship to thyroid hormone parameters. We did not perform in vitro or in vivo functional tests (e.g., gene silencing, overexpression, or inducer-in vitro assays) to experimentally verify the direct involvement of the CDK6 signaling axis in thyroid cells. To clarify: we have revised the discussion section to explicitly reduce the hypothetical claims. The GAS5–CDK6 axis is now explicitly presented as a hypothetical mechanism proposed based on functional studies in other cell types, not as a direct pathway established in our cohort. We have added an explicit statement in the “Limitations and Future Work” section emphasizing the need for future mechanistic studies (e.g., Cell line experiments were used to confirm the direct biological pathway between GAS5, CDK6, and thyroid hormone synthesis.

 Comment 2: "The sample size is small, which weakens the strength of the biomarkers. Try creating demographic subgroups (i.e., distinguishing between males and females as a demographic criterion) to strengthen your findings."

 Author's Response: We agree with the reviewer that the sample size is small, consisting of 30 patients and 20 healthy individuals. This was explicitly stated in the limitations section, and we recommend that future studies be more comprehensive in terms of sample size. However, for gene expression measurement, we believe this may be sufficient or a starting point for future research.

 Comment 3: "The authors conclude that GAS5 is an early biomarker without using the ROC curve, area under the curve (AUC), or sensitivity testing." If you need to state that GAS5 is a biomarker, you must provide an ROC curve analysis and a statement of sensitivity and specificity.

 Author's Response:

 We performed the ROC test and found high diagnostic power for GAS5 in relation to the pathological condition. The table and figure have been added in the revised version.

 Comment 4: "There are spelling errors throughout the manuscript, such as writing 'GADPH' instead of 'GAPDH'."

 Author's Response:

 We apologize for these typographical errors. The entire manuscript has undergone thorough proofreading, line by line. All spelling and formatting errors have been corrected, including correcting 'GADPH' to 'GAPDH' throughout the text and tables.
